# Predicting the Severity of Acute Pain after Cesarean Delivery: A Narrative Review

**DOI:** 10.1007/s11916-024-01301-y

**Published:** 2024-07-23

**Authors:** Lisa Sangkum, Theerawat Chalacheewa, Choosak Tunprasit, Phisut Lavanrattanakul, Henry Liu

**Affiliations:** 1 Department of Anesthesiology, Faculty of Medicine, Ramathibodi Hospital Mahidol University, Bangkok, 10400 Thailand; 2 Department of Anesthesiology and Critical Care, Perelman School of Medicine, University of Pennsylvania, 3400 Spruce Street, Philadelphia, PA 19104 USA

**Keywords:** Cesarean section, Cesarean delivery, Preoperative pain risk assessment, Screening questionare

## Abstract

**Purpose of the Review:**

Cesarean delivery is one of the most common surgical procedures performed worldwide. Approximately 28–78% of the patients have reported experiencing severe pain after Cesarean delivery, which is associated with adverse outcomes. Current analgesic management strategies employ a one-size-fits-all approach, which may not be suitable for all post-Cesarean patients. Our ongoing research and the purpose of this review are focusing on preoperative risk assessment to identify patients at risk of severe pain or needing higher doses of opioid or other analgesics.

**Recent Findings:**

Recent clinical investigations have found that by utilizing the demographic and psychological evaluations, screening tests, quantitative sensory testing, and assessment of response to local anesthetic infiltration, clinicians were potentially able to stratify the risks for severe post-cesarean pain. Several modalities demonstrated significant correlations with pain outcomes, although most of these correlations were weak to modest. Since consensus statement regarding predicting post-CD pain control are still lacking, these correlations can be clinically helpful.

**Summary:**

It is possible to identify patients at high risk of developing severe acute pain after cesarean section by preoperative demographic data, screening questionnaires, or other tools. Further studies are needed to identify additional variables or screening tools for more accurate prediction and investigate whether personalized analgesic regimens can lead to improved analgesic outcomes.

## Introduction

Cesarean delivery (CD) is one of the most common surgical procedures performed around the world. On average about 21.1% women gave birth by CD globally, ranging from 5% in sub-Saharan African region to 42.8% in Latin America and the Caribbeans. The rates of CD have been rising in almost all regions since 1990, with about 1.8 million cesarean procedures annually [1]. Even with widespread use of multimodal analgesia in recent decades, severe acute pain after CD has been reported in 28-78% of the patients [2, 3]. CD ranks the ninth for pain severity among 179 different surgical procedures [4]. Severe acute post-cesarean pain is associated with greater opioid usage, delayed functional recovery [5], and increased risk of postpartum depression [6], all of which may negatively impact the ability to care for her baby and breastfeeding success [7]. Also, it is at an increased risk of persistent postsurgical pain for months after CD and may take opioids after hospital discharge [8].

Analgesic management for post-CD analgesia seemingly adopts a one-size-fits-all approach, which may not be suitable to all CD patient. Komatsu et al. conducted a prospective cohort study in patient who underwent vaginal and CD and these patients were followed up daily after discharge until opioid-free and pain-free recovery. The results showed that the upper 20th percentile of patients experienced a significantly greater pain burden than the remainder [9]. These finding suggested that a one-size-fits all approach may not appropriate for all parturient. The gap between available knowledge and inadequate pain therapy may lead to inconsistent analgesic responses among individual patients and some unsatisfactory patients who underwent CD. This narrative review, therefore, aims at reviewing the utility of various tools to predict the severity of acute pain after CD. Since this may help identification of those patients who are at more risk for postoperative pain and personalized approach of analgesic protocol may be needed.

## Preoperative pain Screening test and Severity of Acute pain and Opioid Consumption after CD

There were several techniques being used as preoperative testing tools in women undergoing cesarean delivery, including preoperative demographic data and psychometric evaluation, quantitative sensory testing (QST), and local anesthetic infiltration.

## Preoperative Demographic data/psychometric Evaluations

In general surgical population, preoperative prognosis factors for poor acute postoperative pain control were studied and found that younger age (OR 1.18), female gender (OR 1.29), higher body mass index (OR 1.02), history of smoking (OR 1.33), depressive symptoms (OR 1.71), anxiety (OR 1.22), sleep difficulties (OR 2.32), and presence of preoperative pain (OR 1.21) or using analgesics (OR 1.54) were significant predictors of poor postoperative pain control [10].

In CD population, the preexisting anxiety (OR 1.93), history of chronic pain (OR 4.12), current tobacco use (OR 2.52), previous CD (OR 1.54), Pfannenstiel incision (OR 3.2), absence of regional anesthesia (OR 3.7) and received intraoperative intravenous ketamine or fentanyl (OR 1.56) were significantly associated with moderate-severe postoperative pain after CD [3, 11, 12].

Preexisting chronic pain has been described in previous studies as the existence of pain as a yes/no answer [13, 14] or by numeric rating scale pain measurement [15, 16], and information regarding the influence of specific chronic pain conditions (e.g., fibromyalgia, migraine, dysmenorrhea). Despite the ongoing debate regarding the precise definition of chronic pain, patients with pre-existing chronic pain conditions often present with symptoms and comorbidities, such as anxiety, depression, poor physical conditioning, and opioid tolerance. Consequently, these patients may experience increased intensity and prolonged duration of postoperative pain.

Also, psychological personalities and pain catastrophizing levels on postoperative analgesia have been investigated. The retrospective cohort study of 2,228 CD patients revealed that patients with anxiety disorders had a higher average pain score (Numeric Rating Scale: 3.9 vs. 3.5, *p* < 0.001) and required more opioids (morphine equivalents used: 110 vs. 102 mg, *p* < 0.001) [17]. Ren et al. conducted a prospective cohort study enrolled 778 pregnant women demonstrated that personality traits such as psychoticism and neurocriticism (OR = 1.43, 95% CI = 1.104–1.844), as well as pain catastrophizing (OR = 7.72, 95% CI = 0.657–0.783), were significant factors influencing post-cesarean analgesia [18]. Additionally, psychosocial factors like being unpartnered or unemployed were linked to higher pain scores post-delivery. These results indicate that addressing psychological personality and social support could potentially improve the post-delivery pain experience.

Various preoperative questionnaires have also been investigated as a tool for predicting post-cesarean pain and analgesic consumption. These included the state anxiety inventory (40-itmes), somatosensory amplification scale score (10-items) [19], pain catastrophizing scale (13-itmes), Pittsburgh sleep quality index (19-items), hospital anxiety and depression scale (14-itmes), patient reported outcomes measurement information system anxiety scale and Eysenck personality questionnaire revised short scale (23-items). Nevertheless, these questionnaires are time-consuming and yield only weak to moderate correlations when it comes to predicting postoperative pain. As such, they pose challenges for application in everyday clinical practice. (Table 1)

**Table 1:** Preoperative screening tools and post-cesarean pain outcomes and analgesic requirement

Study	Questionnaire	Anesthesia technique	Correlation with 24-h postoperative pain scores	Correlation with postoperative analgesic requirement
Pan PH et al. 2006 [20] (*n* = 34)	- State Trait Anxiety Inventory (STAI)	Spinal anesthesia	- STAI was not correlated with pain at rest or on movement following cesarean delivery.	- STAI was correlated with analgesic requirement in recovery room (*r* = 0.39) and total analgesic consumption (*r* = 0.40).
Strulov L et al. 2007 [21] (*n* = 45)	- Pain Catastrophizing Scale (PCD)	Spinal anesthesia	- PCD score was correlated with pain scores on post-cesarean day 2 (*r* = 0.139, *p* = 0.021) but not for post-cesarean day 1.	- PCD score was not correlated with postoperative analgesic requirement.
Gorkem U, et al. 2016 [19] (*n* = 80)	- State Trait Anxiety Inventory (STAI) - Trait anxiety score - Somatosensory Amplification Scale Score	Spinal anesthesia	- STAI showed significant correlation with VAS score ≥ 4 cm within 18 h: adjusted OR = 1.1 (1.0-1.2), *p* = 0.01 - Trait anxiety score and Somatosensory Amplification Scale Score were not correlated with VAS score ≥ 4 cm within 18 h.	- STAI showed significant correlation with pethidine requirement within 18 h after cesarean delivery: adjusted OR = 1.1 (1.0-1.2), *p* = 0.006 - Trait anxiety score and Somatosensory Amplification Scale Score were not correlated with pethidine requirement within 18 h after cesarean delivery.
Borges NC et al. 2016 [3] (*n* = 1,062)	- Hospital Anxiety and depression Scale (HADS)	Spinal anesthesia	- Preoperative anxiety score was associated with moderate-severe pain (OR 1.68 (1.16–2.2), *p* = 0.004)	- Not applicable
Orbach-Zinger et al. 2017 [22] (*n* = 245)	- Pittsburgh Sleep Quality Index (PSQI)	Spinal anesthesia	- Poor sleep quality was associated with increased peak pain score during movement (OR 2.64 95% CI 1.2–6.0; *p* = 0.02) - The sensitivity of PSQI to predict severe peak pain score upon movement during first 24 h. was 82.2% - Pain at rest or with movement, average uterine cramping, peak pain at rest or peak uterine cramping were comparable between patient with poor and good sleep quality group	- Women with poor sleep quality required greater additional analgesic medication for breakthrough pain in the first 24 h. after caesarean delivery

The preoperatively applied simple rating questionnaire, which allows patients to rate their anxiety, anticipated pain, analgesic needs, and pain thresholds, have been proposed and it showed to predict the intensity of post-CD pain [23, 24]. Pan et al. found that the three screening questions demonstrated a sensitivity and specificity of 0.68 and 0.67, respectively, for identifying patients in the top 20th percentile for post-CD pain. Another study by Carvalho et al. showed that three slightly different questionnaires were able to predict 45% of the variability of post-CD pain and 21% of the variance in opioid consumption. (Table 2) However, the utility of the questionnaire may require more in other institutions/ setting and focus in low-risk group who receiving the same interventions as those predicted as high-risk.

**Table 2:** Preoperative three simple rating questions to screen post-CD pain

Study	Questionnaire	Anesthesia technique	Correlation with 24 h postoperative pain scores	Correlation with postoperative analgesic requirement
Pan et al., 2013 [23] (*N* = 192)	**Three simple questions** - How anxious are you about your upcoming? (VAS 0-100) - How much pain do you anticipate experiencing after your upcoming surgery? (VAS 0-100) - How much pain medication do you anticipate needing after your upcoming surgery (0–5)	**Spinal anesthesia** - IT fentanyl 15–20 mcg + MO 150–200 mcg **Postoperative pain** - Ibuprofen 800 mg po q 6 h. - Oxycodone/ Acetaminophen for breakthrough pain	- Anxious (*r* = 0.24) - Anticipated pain (*r* = 0.33) - Anticipated pain medication (*r* = 0.33)	- No significant correlation between simple screening tool and analgesic requirement
Carvalho et al., 2016 [24] (*N* = 50)	**Psychological questionnaire** - How much pain do you expect to experience after your surgery on a pain scale 0–10? - What do you expect your analgesic requirements will be after surgery? (0–10) - At what point on a pain scale of 0–10 would you likely request postoperative pain relief? (0–10)	**Spinal anesthesia** - IT fentanyl 10 mcg + morphine 100 mcg **Postoperative pain** - Ibuprofen 800 mg po q 6 h. for 48 h. - Oxycodone/hydrocodone 5 mg with acetaminophen 325 mg po for pain ≤ 4/10 - Oxycodone/hydrocodone 10 mg with acetaminophen 650 mg po for pain > 4/10 - Intravenous morphine 4 mg for severe acute pain	- Expected postoperative pain (*r* = 0.349) - Anticipated analgesic threshold (*r* = 0.032) - Perceived analgesic need (*r* = 0.169) - Overall, three-simple questionnaire (*r* = 0.447)	- Expected postoperative pain (*r* = 0.263) - Anticipated analgesic threshold (*r*= -0.349) - Perceived analgesic need (*r* = 0.313) - Overall, three-simple questionnaire (*r* = 0.212)
Chan JJI, et al. 2020 [25] (*n* = 217)	**Three simple questions** - How anxious are you about your upcoming? (VAS 0-100) - How much pain do you anticipate experiencing after your upcoming surgery? (VAS 0-100) - How much pain medication do you anticipate needing after your upcoming surgery (0–5) **Others questionnaire** - Hospital Anxiety and Depression Scale (HADS) - Pain Catastrophizing Scale (PCD)	**Spinal anesthesia or combined spinal epidural anesthesia.** - IT fentanyl 15 mcg + MO 100 mcg **Postoperative pain** - Paracetamol and mefenamic acid were administered to patients after cesarean delivery - Tramadol would be administered as per patient request for excess pain.	- Anxious was significantly associated with moderate-to-severe acute pain at 24 h. (OR = 1.02, 95% CI = 1.01–1.04, *p* = 0.007) - Anticipated pain (OR = 1.02, 95% CI = 1.00–1.04, *p* = 0.055) and anticipated pain medication (OR = 0.97, 95% CI = 0.66–1.43, *p* = 0.88) was not associated with moderate-to-severe acute pain at 24 h. - Preoperative HADS depression (OR = 0.99, 95% CI = 0.89–1.10, *P* = 0.81) and HADS anxiety (OR = 1.08, 95% CI = 0.98–1.18, *p* = 0.13) did not show any significant association with acute post-cesarean pain at 24 h. - Total pain catastrophizing scores (OR = 1.04, 95% CI = 1.00–1.07, *p* = 0.024) were associated with moderate-to-severe acute post-cesarean pain at 24 h	- Not applicable
Gupta D et al. 2023 [26] (*n* = 150)	**Three item questionnaires** - How anxious are you about your upcoming? (VAS 0-100) - How much pain do you anticipate experiencing after your upcoming surgery? (VAS 0-100) - How much pain medication do you anticipate needing after your upcoming surgery (0–5)	**Spinal anesthesia** - IT fentanyl 25 mcg **Postoperative pain** - Paracetamol 1 gm IV q 8 h. - Diclofenac 75 mg IV q 8 h. - Tramadol 1 mg/kg IV prn for VNPS ≥ 3	**Pain at rest** - Anticipated pain (*r* = 0.124, *p* = 0.15) - Anxious (*r* = 0.185, *p* = 0.03) - Anticipated pain medication (*r* = 0.24, *p* = 0.005) - Overall, three item questionnaires (*r* = 0.225, *p* = 0.008) - No significant correlation between three item questionnaires and evoked pain.	- Not applicable

Although there is currently no consensus on preoperative demographic, psychometric evaluation or screening tools for post-CD pain, these demographic characteristics and preoperative screening tools may assist clinicians in determining high-risk for severe pain patients and tailoring analgesic regimens to achieve individualized pain management.

## Preoperative pain Stimulation Methods

QST defined as quantifiable mechanical (pressure), thermal (warm, heat pain) or electrical stimuli, was used in nearly all the studies.

### Pressure Stimulation

Pressure stimulation is usually used to determine pain threshold and pain tolerance. A probe is applied to the pulp of the middle finger, and patients are asked to indicate when they first feel pain (pain threshold) and when they can no longer tolerate the pain (pain tolerance) [27, 28]. In three studies, pressure-induced pain tolerance and threshold were determined using a digital algometer. The results of these studies showed a weak correlation between pressure pain threshold/tolerance and postoperative pain outcomes after CD [2, 27, 28] (Table 3). Another technique using a 180 g Von Frey filament to measure mechanical temporal summation (mTS), a dynamic quantitative sensory testing. The result showed that mTS was significantly correlated with 24-hrs VAS on movement but not correlated with 24 h VAS or VAS at rest [2]. Another study by Ortner et al. using the 180 g Von Frey filament to evaluate scar hyperalgesia around the area of prior CD scars and calculate scar hyperalgesia (SHA) index. The results showed that preoperative SHA index was correlated with postoperative pain severity and wound hyperalgesia at 48 h [29]. Contrary to the results found in Chan et al. study, the mTS was not associated with moderate-severe acute pain after CD [25].

### Thermal Stimulation

A hand-held device with a contact probe that can generate a range of temperatures was used. The temperature at which the heat stimulus became painful was determined as the pain threshold, and pain scores in response to the heat stimulus were recorded as suprathreshold stimulus. Three studies were conducted to evaluate the correlation between thermal stimulation and post-CD pain outcomes. The results suggested a weak to moderate correlation [20, 21, 30] (Table 3).

### Electrical Stimulation

An electrocutaneous stimulation device was used to generate electrical discharges to determine pain/sensation thresholds. Preoperative electric pain/ sensation thresholds were inversely correlated with postoperative pain scores and analgesic requirement. The literature showed that preoperative electric QST can predict some of post-CD pain. One study reported weak correlation [31] (Table 3). However, another study reported moderate-strong correlation [32]. Some studies evaluating experimental models of pain threshold/tolerance with pressure, electric, and thermal stimuli all demonstrated some correlation with postoperative pain outcomes after CD. However, most of the findings were not consistent, and the correlations were weak to modest in most of the studies. Moreover, these tests required approximately 120 min and additional personnel equipment, and training, making it impractical for routine clinical setting. Therefore, the utility of QSTs is still limited in daily practice. Future research may need to incorporate simpler, clinically more applicable prediction tests in a decision algorithm for analgesia after CD.

**Table 3:** Preoperative quantitative sensory testing and postoperative pain outcomes and analgesic requirement

Study	Type of stimulation	Anesthetic technique	Correlation with postoperative pain outcomes	Correlation with postoperative analgesic requirement
Guevara J et al. 2021 [2] (*n* = 195)	- Pressure algometry: PainTest^™^ FPX 25 digital algometer (Wagner Instruments, Greenwich, CT, USA) - Mechanical temporal summation (mTS)	Spinal anesthesia	- Preoperative pressure algometry was correlated with 48-hr VAS on movement (*r*= -0.22, *p* = 0.01) - No correlation was found between preoperative pressure algometry and VAS at rest. - mTS was correlated with 48-hr VAS on movement but not for 24 h VAS - No correlation was found between mTS and VAS at rest.	- Preoperative pressure algometry correlated with 24–48 h. morphine requirement (OR 0.87 (0.76 to 0.99), *p* = 0.03) but not for the first 24 h. after cesarean delivery.
Buhagiar L et al. 2011 [27] (*n* = 65)	- Electrical pain assessment (PainMatcher^®^) - Pressure pain assessment (digital algometry) (PainTest™FPN 100 and PainTest™ FPX25 Algometer) - Determine pain threshold & tolerance	General anesthesia or spinal anesthesia	- Electrical pain threshold correlated with NRS at 6 h. (*r* = -0.26) and 24 h. (*r* = -0.23) but not NRS at 12–24 h. - Pressure pain tolerance correlated with NRS at 6 h. but not NRS at 12, 24, or 48 h. - No significant correlation between pressure pain threshold and pain scores at 6, 12, 24, or 48 h.	- Electrical pain threshold correlated with first 48-hrs acetaminophen consumption (*r* = -0.33) but electrical pain tolerance had no correlation with acetaminophen consumption. - No significant correlation between pressure pain tolerance or pressure pain threshold and acetaminophen consumption in the first 48 h.
Buhagirar L et al. [28] (*n* = 20)	- Electrical pain assessment (PainMatcher^®^) - Pressure pain assessment (digital algometer) (PainTest™FPN 100 and PainTest™ FPX25 Algometer) - Determine pain threshold & tolerance	General anesthesia or spinal anesthesia	- Electrical pain threshold correlated with NRS at 6 h. (*r* = -0.48) but not NRS at 12, 24, or 48 h. - No significant correlation between pressure pain threshold and NRS.	- Electrical pain threshold correlated with 48 h. morphine requirement (*r* = -0.45) - Pressure pain threshold correlated with 48 h. morphine requirement (*r* = -0.41) - Pressure pain tolerance correlated with 48 h. morphine requirement (*r* = -0.44)
Granot M et al. 2003 [30] (*n* = 58)	- Thermal pain threshold and thermal suprathreshold pain (Thermal Sensory Analyzer (TSA-2001; Medoc, Ramat-Yisai, Israel)	Epidural anesthesia	- Preoperative suprathreshold pain scores at 48ºc correlated with post-cesarean pain at rest (*r* = 0.492, *p* = 0.003), and with during activity (*r* = 0.527, *p* = 0.003) on the morning after cesarean delivery - No significant correlation was found between the preoperative pain threshold and post-cesarean section pain scores for either condition	Not applicable
Pan PH et al. 2006 [20] (*n* = 34)	- Thermal pain threshold and suprathreshold pain (Thermal Sensory Analyzer (Model TSA II; Medoc Inc., Ramat Yishai, Israel)-	Spinal anesthesia	- Preoperative thermal pain threshold correlated with pain at rest (*r* = -0.036) and pain on movement (*r* = -0.42) at 20–24 h after cesarean delivery - Preoperative suprathreshold thermal pain intensity and unpleasantness correlated with pain at rest (*r* = 0.45), and pain on movement (*r* = 0.40) at 20–24 h after cesarean delivery	- Preoperative thermal pain threshold correlated with analgesic requirement in recovery room (*r* = -0.43) but not for total analgesic requirement. - Preoperative suprathreshold thermal pain intensity and unpleasantness correlated with analgesic requirement in recovery room (*r* = 0.48) and total analgesic consumption (*r* = 0.43)
Strulov L et al. 2007 [21] (*n* = 47)	- Thermal pain threshold and suprathreshold pain (Thermal Sensory Analyzer (TSA-2001, Medoc, Ramat- Yishai, Israel)	Spinal anesthesia	- Preoperative thermal stimulus visual analog scale at 10, 20, 40 and 55 s during heat stimulus correlated with post-cesarean pain day 1 (*r* = 0.393, *p* = 0.008)	- No significant association was observed between preoperative response to heat stimuli and the patient’s analgesic consumption in the obstetrical ward.
Wilder-Smith CH et al. 2003 [31] (*n* = 120)	- Electrical sensory testing for sensation and pain tolerance thresholds (using nerve stimulator)	Spinal anesthesia	- Preoperative sensation correlated with pain scores 2 h. after surgery (*r* = -0.22, *p* = 0.02) - Preoperative pain thresholds correlated with pain scores 2 h. after surgery (*r* = -0.26, *p* = 0.006)	- No significant correlation between pain thresholds and time to first analgesic requirement
Nielsen PR et al. 2007 [32] (*n* = 39)	- Electrocutaneous stimulation (pain matcher; Cefar medical AB, Lund, Sweden)	Spinal anesthesia	- Preoperative electrical pain threshold was correlated with postoperative pain score at rest (*r* = -0.65 (95% CI: − 0.30 to − 0.75), *p* < 0.01], and at mobilization [*r* = − 0.52 (95% CI: − 0.23 to − 0.72), *p* < 0.01]	- No significant correlation between electrical sensory threshold and the post-operative need for supple- mental analgesics
Chan JJI, et al. 2020 [25] (*n* = 217)	- Mechanical temporal summation using a 180-gram von Frey filament	Spinal anesthesia or combined spinal epidural	- An evoked mTS (pinprick differences > 0) (OR = 0.89, 95% CI = 0.45–1.76, *P* = 0.7274), pain scores at rest (OR = 1.07, 95% CI = 0.78–1.50, *p* = 0.6761), and pain scores with movement (OR = 1.05, 95% CI = 0.78–1.43, *p* = 0.7590) were also not significantly associated with moderate-to severe acute post-cesarean pain at 24 h.	Not applicable
Ortner CM et al. 2013 [29] (*n* = 163)	- Mechanical temporal summation to evaluate scar hyperalgesia (SHA) index	Spinal anesthesia	- SHA index was correlated with post-operative pain severity (*r* = 0.25, *p* < 0.002) and wound hyperalgesia at 48 h (*r* = 0.608, *p* < 0.001) - Severe pain (visual analogue pain scale > 7) was predicted with a sensitivity and specificity of 60% and 62%, respectively	- No significant correlation between SHA index and analgesic requirement

### Local Anesthetic Infiltration

Local anesthetic skin infiltration is usually performed as part of standard procedure when placing spinal or epidural anesthesia for women who undergoing CD. Regarding the correlation between pain during local anesthetic infiltration and post-cesarean pain, Orbach-Zinger et al. found that pain scores on local anesthetic infiltration were able to predict severe acute postoperative pain with good correlation (*r* = 0.529, *p* < 0.001), sensitivity 91.6% and specificity 93.3% [22]. However, Gupta et al. showed the weak correlation of pain intensity upon local infiltration and postoperative pain (*r* = 0.195) [26]. Another study by Chan et al. found no association between increased pain score upon local anesthetic injection and acute post-cesarean pain (unadjusted OR = 1.10, 95% CI 0.95–1.27, *p* = 0.21) [25]. Therefore, further study may need to evaluate the utility of this method for predicting post- CD pain.

## Combination of Multiple Modalities

There are 4 studies investigated multiple modalities to predict post-CD pain, using questionnaires as QST, and suprathreshold pain intensity and unpleasantness [2, 20, 27, 28]. For example, a 3-item questionnaire (3-IQ) used in combination with mechanical temporal summation and pressure algometry had area under the ROC curve values of 0.64 and 0.67, respectively, to predict VAS scores on movement greater than or equal to 70 [2]. The results showed that a combination of factors would be more accurate in predicting post-CD than a single modality. However, the combination of factors may not always be beneficial for prediction of postoperative pain after CD. The results showed that combined 3-itme question did not enhance the predictive value of VAS scores (correlation coefficient of all 3-IQ vs. anticipated pain: 0.24 vs. 0.25) [2]. Therefore, further studies may need to determine which factors or how many should be utilized to predict postoperative pain following CD. Additionally, these factors should be easily implementable in clinical practice while ensuring the highest level of accuracy.

## Conclusions

Postoperative pain following CD poses a significant challenge that warrants future improvement. Acute postoperative pain serves as a strong independent predictor of adverse outcomes and chronic pain following CD. Recent research suggested the one-size-fits-all approach may not be optimal for the entire post-cesarean population. While consensus statements on predicting post-cesarean pain control are lacking, preoperative demographic data and screening tools may help identify patients at higher risk of developing severe acute pain. By targeting these high-risk patients with tailored individualized interventions, outcomes may be enhanced. Future studies should aim to identify additional variables or screening tools for better prediction of post-cesarean pain and investigate whether personalized analgesic regimens can lead to improved outcomes in these individuals.

## Data Availability

No datasets were generated or analysed during the current study.
